# Psychometric Properties of the Persian Version of Screening for Somatic Symptom Disorders-7(SOMS-7)

**Published:** 2018-10

**Authors:** Amrollah Ebrahimi, Winfried Rief, Peyman Mirshahzadeh, Hamid Afshar Zanjani, Hamid Nasiri Dehsorkhi, Hamidreza Roohafza, Awat Feizi, Peyman Adibi

**Affiliations:** 1 Behavioral Sciences Research Center, Department of Psychiatry, Isfahan University of Medical Sciences, Isfahan, Iran.; 2 Department of Clinical Psychology and Psychotherapy, Philipps-University of Marburg, Marburg, Germany.; 3 Psychosomatic Research Center, Department of Psychiatry, Isfahan University of Medical Sciences, Isfahan, Iran.; 4 Cardiac Rehabilitation Research Center, Cardiovascular Research Institute, Isfahan University of Medical Sciences, Isfahan, Iran.; 5 Department of Biostatistics and Epidemiology, School of Health, Isfahan University of Medical Sciences, Isfahan, Iran.; 6 Integrative Functional Gastroenterology Research Center, Isfahan University of Medical Sciences, Isfahan, Iran.

**Keywords:** *Persian Version*, *Reliability*, *Screening for Somatic Symptom Disorders*, *SOMS-7*, *Validity*

## Abstract

**Objective:** Somatic symptoms are one of the most prevalent complaints in both psychiatric and general population, and validated scales are required to assess these problems. The present study was conducted to determine psychometric properties of the Persian version of Screening for Somatic Symptom Disorders-7(SOMS-7) in an Iranian population.

**Method** **:** This was a multi centric comprehensive study conducted in Psychosomatic Research Center of Isfahan University of Medical Sciences in collaboration with Department of Clinical Psychology of Philipp University of Marburg, Germany. This part of the study includes 100 patients with anxiety/mood disorders and 291 healthy individuals. All participants completed the Patient Health Questionnaire (PHQ-15) and Screening for Somatic symptom disorders 7(SOMS-7). Data were analyzed by Pearson and Spearman correlation coefficient, factor analysis, independent t test, and discriminant analysis using SPSS-20 software.

**Results: **Reliability coefficient based on Cronbach’s alpha was 0.92 and 0.94 (clinical vs. healthy sample). Validity index of the SOMS according to correlation between factor 1 and 2 with PHQ somatic subscale was. 51 and. 59, respectively. Score of 15.5 as cut-off point was accompanied with sensitivity of 77% and specificity of 66%. Factor analysis extracted 2 factors in patients and 4 factors in healthy population.

**Conclusion: **Findings of this study indicated that the Persian version of SOMS-7 has appropriate reliability and validity for the assessment of somatic symptoms disorder and evaluation of treatment effects in these patients.

Somatoform disorders, also called somatic symptoms disorder (SSD) in the Diagnostic and Statistical Manual of Mental Disorders –fifth edition (DSM-5), are a prevalent broad group of diseases that includes somatic signs and symptoms that cannot be sufficiently explained by medical. These diseases cause unreasonable visits to primary care centers and inappropriate care giving (1). 

This diagnostic category for some of them includes physical symptoms that have lasted at least 6 months and led to disruption of daily life. These intrusive thoughts are about a particular illness or excessive concern about a disease that may cause overspending of energy and time to keep track of these symptoms (2). Patients affected by somatic symptoms are deeply concerned about the slightest problems in their body and interpret them in a negative manner. 

The etiology of this disorder has been explained through biopsychosocial approach. Hypersensitivity to pain and/or proprioception is mentioned for biologic factors, history of familial violence, or child abuse for social factors, and over-attention gained due to an illness for psychological factors (3).

This disorder may be accompanied with other disorders such as depression, anxiety, or personality disorders. Also, it should be differentiated from transient physical symptoms, fear with physical symptoms induced in anxiety disorders, psychotic disorders, and withdrawal signs (4). 

The prevalence of somatic symptom disorder is estimated to be11%- 21% in youths, 10%-20% in adults, and 1.5%-13% in the elderly. These patients receive high rates of medical care that impose heavy economic burden on the community. 

Thus, a special strategy is needed to reduce signs and symptoms of these patients to lower their health care costs (5). 

Diagnosticians are dissatisfied about categorization and diagnosis of this disorder, as most of these patients are generally categorized in undifferentiated groups, in particular in ICD-10. One problem in this context is that the diagnostic criteria for these disorders only focus on symptoms, while psychological and psychophysiological processes are neglected (6). Thus, an appropriate tool is needed to screen and assess this disorder. In fact, a valid and reliable questionnaire in which all aspects of this disorder are considered is necessary. Also, the questionnaire should be reliable and valid. To diagnose and determine SSD severity, various scales, including Somatization Scale of the Symptom Checklist and SCL-90R or MMPI, have been used (7). SOMS has numerous advantages compared to other tools that had been used for evaluation of somatoform disorders (SFD). This questionnaire assesses extensive somatic symptoms and is directly in accordance with ICD-10 and DSM-5. Another superiority of SOMS is the formation of questions in which the patient only reports symptoms with no obvious underlying organic disorders (8).

In 2014, a cross sectional study was conducted in Denmark for somatic symptom disorder screening. The questionnaire’s validity and reliability were evaluated. Its primary 30-assessed items were reduced to 25 items and satisfactory psychometric results were obtained (9). Another study with 98 items was conducted in Germany based on the somatic symptom disorder-B criteria scale (SSD-12). SSD-12 reliability and validity were also assessed (10). Considering the prevalence of psychosomatic disorders, especially somatic symptoms disorder in Iran and the need for screening and identifying these disorders in primary care setting and general population, designing appropriate tools are highly important. Unfortunately, there are no adopted or validated questionnaires specialized to assess somatoform disorder or somatic symptoms disorder in Iran.

Thus, the present study was conducted to prepare, localize, and determine the psychometric properties of SOMS-7 (validity, reliability, factor structure) and determine the clinical cut-off point accompanied with sensitivity and specificity in healthy population and in patients with anxiety/mood disorder in Iran.

## Materials and Methods

This cross sectional methodological study was conducted on 391 participants, including 100 patients with anxiety/mood disorders and 291 healthy participants, in Isfahan Province, Iran, in 2016. Based on a calling plan, all accessible patients with anxiety disorders who referred to psychiatric clinics and private clinics were included. Healthy participants were selected through online call, governmental and private centers, and students. The non-clinical samples were matched according to the demographic characteristics, such as age, gender, and education. Moreover, the total sample size included 1216 participants in the original project.

***Inclusion Criteria for the Patients***

Persian male and female patients, aged 18-60 years, with the education level of at least reading and writing, who had one of the anxiety disorders (generalized anxiety disorder, panic, and phobias) and were diagnosed by psychiatrists according to DSM-5 were included in the study.

Patients with other mental disorders, such as psychosis, schizoaffective, bipolar disorder, and Substance or Medication Induced Anxiety Disorder, major cognitive problems, non-Persian speaking, and lack of desire to continue cooperation were excluded.

***Criteria for the General Population***

Inclusion criteria were Persian speaking, education level of at least reading and writing, willingness to participate, and age 18-60 years. Presence of a mental disorder based on an interview, chronic physical illness (physical disabilities, mental, cognitive, cardiovascular and neurological disorders, etc.) and reluctance for cooperation were exclusion criteria for the control group.

This study was designed based on a Memorandum of Understanding between Psychosomatic Research Center of Isfahan University of Medical Sciences (Ethics Committee code: IRMUI.REC1394.1.73) and Philips University of Marburg, Germany (Ethics Committee code: 2014-8-K). This study was part of a comprehensive research project to provide appropriate tools to evaluate somatoform symptoms and assess prevalence of psychosomatic symptoms in samples of Iranian and German population. Questionnaires were translated into Persian and presented to the expert panel, which included gastroenterologists, psychiatrists, and clinical psychologists, in 3 meetings. Then, the questionnaire was back-translated into English by a bilingual expert with a PhD degree and, then, was sent back for reevaluation. New evaluated English questionnaire was presented to the expert panel again and, accordingly, they made changes in some items and decided on new proper terms. The pre-final version was presented to anxious patients, students, and healthy individuals in a pilot study. During the enforcement, participants' views about items were asked. This version was also presented to 10 clinical psychologists and psychiatrists to assess the items based on the aims of the study and determine the content validity.

Finally, opinions of patients, participants, and specialists were asked and the expert panel reevaluated the results. Finally, the Persian version of SOMS-7 Questionnaire was provided. In the next step, the final questionnaire was presented to gastroenterologists, psychiatrists, and clinical psychologists to evaluate content validity based on the aims of the study. To assess test-retest reliability, 50 persons, including anxiety/mood disorders patients and healthy individuals, were asked to complete the final questionnaire.

***Instruments***

1 (Clinical psychiatric interview with the patients according to the DSM-5 for primary diagnosis

2 (Demographic questionnaire with 28 questions about individual, familial, social, economic, and medical history

3 (SOMS-7 questionnaire: Screening for Somatic Symptom Disorders-7(SOMS-7) is a questionnaire with 53 items designed to evaluate the effects of treatment in patients with somatic symptom disorders. This tool consists of all aspects of somatic symptom disorder and evaluates patients' signs/symptoms in 7 days. Signs/symptoms severity is defined using Likert scale: score of 0 for the least severity and 4 for Maximum severity. SOMS-7 is a new scale that shows 2 different indices including signs/symptoms of somatic symptom disorders and their severity. These 2 indices help differentiate patients with somatic symptom disorders who do not meet the complete criteria compared to those who do. This questionnaire has high reliability and sensitivity reported by Hiller et al. Also, 72-hour test-retest of this questionnaire showed the reliability of 0.85 and validity of 0.75 through self-reported symptoms and clinical interview (7).

4 (Patient Health Questionnaire (PHQ-15): This questionnaire was presented by Kroenke, Spitzer et al. and has 5 subscales, including physical symptoms, anxiety, depression, panic, and eating disorder. It is scored based on a 4-point Likert scale. Internal consistency of PHQ-15 was 0.72 and test-retest value 0.87(11, 12).

***Statistical Analysis***

Descriptive data were reported in mean and standard deviation. To assess reliability and internal consistency, Cronbach's alpha and test-retest with 2-week interval was used. With respect to validity assessment, Pearson correlation coefficient of SOMS-7 scores with that of PHQ-15 was performed. Exploratory factor analysis was used to determine factor structure. Discriminant analysis and the ROC curve were used to determine the discriminant validity and obtain cut-off point and sensitivity and specificity. P-value<0.05 was considered significant.

## Results

This study was conducted on 291 healthy individuals and 100 patients with anxiety/mood disorder in Iran. The mean age of the healthy individuals and patients was 29.6 ±12.0 and 34.2 ±10.2, respectively. In terms of sex, 62.2% of the healthy individuals and 69.2% of the patients were female. Other demographic characteristics are displayed in Table 1.

***Reliability***

Internal consistency based on Cronbach's alpha was 0.94 for the general population and 0.92 for patients. In addition, test-retest was conducted with a 2-week interval, and reliability was 0.86 and 0.70 for the general population and patients, respectively. Cronbach's alpha of factors is presented in Tables 2 and 3.

***Factor Analysis of the Persian Version SOMS-7 Scale in the Healthy Population***

This part of the study consisted of 291 cases who filled in the questionnaire. KMO and Bartlett’s test was used (0.818), and the result ensured the adequacy of sample size for factor analysis. Eigenvalues above 1 was considered. Points in screen plots showed that the 47 items in SOMS-7 questionnaire contain 4 main factors (dimensions). Varimax rotating method was used and based on the Rotated Component Matrix Table, questions loading on each factor were as follow (Table 2):

A) First factors (pain, cardiovascular and respiratory symptoms): Questions 1, 2, 3, 4, 5, 6, 11, 12, 15, 24, 25, 26, 27, 28, 29, 30, 31, 32, 41, and 42. 

B) Second factors (gastrointestinal and urologic symptoms): Questions 2, 7, 8, 9, 10, 12, 13, 14, 16, 17, 18, 19, 20, 33, 36, and 37.

C) Third factors (neurological functioning symptoms): Questions 34, 37, 39, 40, 42, 43, 44, 45, 46, and 47. 

D) Fourth factors (musculoskeletal symptoms): Questions 4, 5, 18, 19, 34, and 35.

Some questions were placed in more than 1 category. This condition occurred because of a high score or high number of patients complaining about symptoms in more than 1 category. Questions 21, 22, and 23 were removed due to weak factor loading.

***Factor analysis of the Persian Version of SOMS-7 Scale among Anxiety/Mood Disorder Patients***

In the present study, there were 100 anxiety/mood disorder patients. KMO and Bartlett’s test indicated sample size adequacy for factor analysis. 

Factor analysis revealed that the 47 questions in SOMS-7 loaded on 2 main factors (dimensions). To rotate these factors, Varimax method was used and based on Rotated Component Matrix Table, questions loading on each factor were as follow (Table 3):

A) First factors (cardiovascular, respiratory and gastrointestinal symptoms): Questions 1, 6, 8, 9, 10, 12, 13, 14, 15, 17, 18, 19, 20, 24, 25, 26, 27, 28, 29, 30, 31, 34, 35, 36, 38, 41, 42, 43, 45, 46, and 47.

B) Second factors (pain, musculoskeletal and neurological symptoms): Questions 2, 3, 4, 5, 7, 8, 11, 14, 16, 20, 21, 22, 23, 32, 33, 35, 37, 39, 40, 43, 44, 45, 46, and 47. 

Some questions were placed in more than 1 factor, which was due to a high score or high number of patients complaining about symptoms in more than 1 category. Questions 8, 14, 20, 35, 43, 45, 46, and 47 were candidates to be removed due to inappropriate factor loading in each scale.

***Validity***

Validity of SOMS-7 was determined through construction, discriminant, and convergent validity assessment. Convergent validity was assessed by considering the correlation of factor analysis scores with subscales of PHQ-9 (Table 4).

Findings in Table 4 reveals that SOMS-7 questionnaire is significantly correlated with PHQ somatic scale. Thus, these findings indicated that SOMS-7 can be a valid scale.

This assessment was also performed for patient groups. Table 5 displays the correlation of SOMS-7 score and PHQ score in patients with anxiety/mood disorder.

Findings of Table 5 show that SOMS-7 questionnaire has a significant correlation with PHQ somatic scale. 

The difference in the mean scores of SOMS-7 of the general population and patients was compared (Table 6). Discriminant analysis and the ROC curve were used to determine distinction validity.

Mean scores of the general population, compared to patients, are presented in Table 1, and showed significant differences, which indicated discrimination validity of this scale.

**Figure 1:**
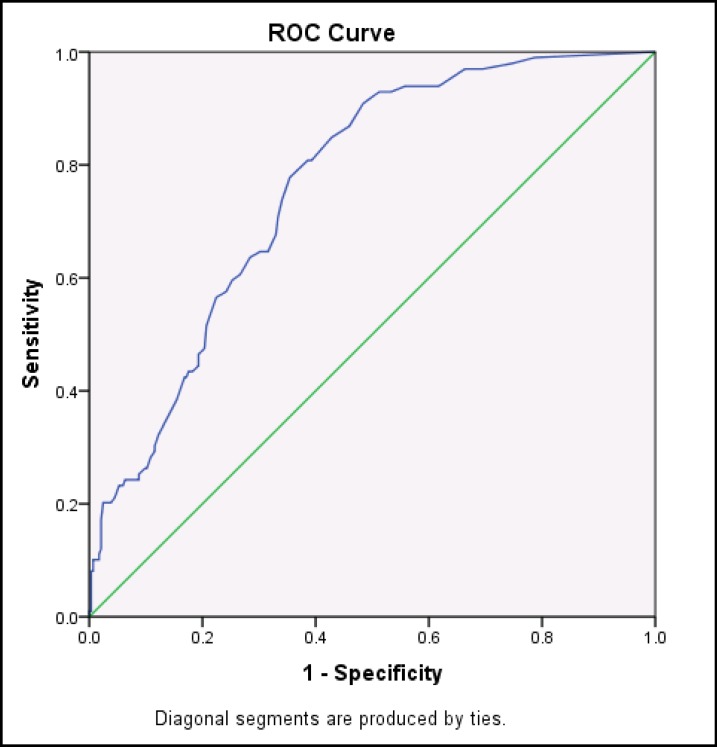
ROC Curve of SOMS-7 Questionnaire for Anxiety/Mood Disorder Patients

ROC Curve (Figure 1) for distinction validity of SOMS-7 questionnaire is presented below (P-value<0.001; CI, 0.713-0.812). The score of ≥ 15.5 from SOMS-7 questionnaire has 77% sensitivity and 66% specificity in the diagnosis of patients with anxiety/mood disorder.

**Table 1:** Descriptive Statistics of Psychometric Properties of the Persian Version of the Patients and the Healthy Sample in Iran and Germany

	**Subclinical Sample**			**Patient Sample**		
	Iran	Germany	Statistical comparison	Iran	Germany	Statistical comparison
N Age (M, SD years)	291 29.6 (12.0)	102 28.0 (9.2)	t (222.5) = 1.38	100 34.2 (10.2)	90 41.0 (13.1)	t(169.6) = 3.92***
Sex (% female)	62.2	79.4	χ² (2) = 17.13***	69.2	60.4	χ² (1) = 1.54
Educational level (%) Primary school Secondary school Diploma Associate degree Bachelor’s degree Master’s degree	1.2 1.2 28.8 6.8 34.0 28.0	52.9 10.8 11.8 24.5	χ² (5) = 29.36***	11.1 6.7 23.3 10.0 35.6 13.3	1.1 26.7 54.4 10.0 2.2 5.6	χ² (5) = 58.72***
Religiousness (%) Believer in religion Merely doing duties Without religious belief	43.0 40.8 16.2	18.2 27.3 54.5	χ² (2) = 55.32***	48.8 39.3 11.9	10.0 53.3 36.7	χ² (2)= 35.4***
Diagnosis (%) Mood Anxiety Mixed				64.8 35.2	77.8 6.1 16.1	
Hospitalization (%) Outpatient treatment (%)				31.7 72.1	53.5 67.4	χ² (1)= 8.3** χ² (1) =. 45
Mental stress (M, SD) PHQ9^a^ GAD7^b^ PHQ15^c^ SOMS7^d^	8.1 (5.3) 5.2 (4.4) 8.6 (4.7) 5.5 (6.1)	6.5 (4.0) 4.1 (2.0) 4.1 (2.0) 1.9 (2.4)	t (238.4) = 3.20** t (358.6) = 8.45*** t (372.1) = 13.20*** t (372.4) = 8.19***	14.0 (6.5) 10.6 (5.8) 11.7 (5.1) 10.5 (8.6)	7.6 (4.9) 3.5 (2.3) 4.8 (3.3) 5.3 (5.8)	t (167.2) = -7.38*** t (115.6) = -10.85*** t (154.7) = 10.80*** t (156.5) = 4.73***

Note. N = Sample size, M = Mean, SD = Standard deviation, t = t value, χ² = Chi-square value,

a Depression score of the PHQ,

b Anxiety score of the PHQ,

c Score for somatoform symptoms of the PHQ,

d Score for somatization symptom count of the SOMS7

* p< 0.05,

** p<0.01,

*** p <0.001.

**Table 2:** Factor Analysis of Screening for Somatic Symptom Disorders-7(SOMS-7) in Healthy Population

**Title of Items**	**Factors**			
	**1**	**2**	**3**	**4**
1. Headache	0.454			
2. Stomachache	0.420	0.410		
3. Lumbago	0.491			
4. Pain in joints	0.433			0.671
5. Pain in hand, leg	0.471			0.670
6. Pain in chest	0.662			
7. Pain in anus		0.361		
8. Pain during sexual intercourse		0.221		
9. Pain during urination		0.346		
10. Nausea		0.556		
11. Meteorism	0.533			
12. Stomachache Anxiety	0.526	0.399		
13. Vomiting(not in pregnancy)		0.530		
14. Belching		0.410		
15. Hiccups Burning in chest	0.490			
16. Inability to digest some foods		0.400		
17. In appetence		0.526		
18. Sour taste in mouth		0.394		0.399
19. Mouth dryness		0.438		0.450
20. Repeated diarrhea		0.650		
21. Exit of fluids, moisture from anus		0.287		
22. Repeated urination		0.265		
23. Repeated Defecation		0.239		
24. Palpitations	0.825			
25. Discomfort in Heart	0.776			
26. Sweating(hot, cold)	0.638			
27. Hot flashes	0.709			
28. Shortness of Breath without activity	0.702			
29. Gasping for Breath	0.715			
30. Excess Fatigue without activity	0.542			
31. Spots, color changes in skin	0.464			
32. Frigidity	0.480			
33. Discomfort in Genital organs		0.316		
34. Imbalance of movements			0.450	0.411
35. Muscular paralysis				0.497
36. Trouble swallowing		0.324		
37. Loss of voice			0.471	
38. Cease to urinate		0.371		
39. Imagining (seeing/ hearing unreal things)			0.385	
40. Loss of sense of touch or pain			0.451	
41. Shivering	.520			
42. Diplopia	.401		0.387	
43. Temporary Blindness			0.798	
44. Temporary loss of hearing			0.829	
45. Convulsion Attacks			0.835	
46. Amnesia			0.375	
47. Losing consciousness			0.551	
Number	254	233	274	272
Cronbach's alpha	0.923	0.836	0.770	0.820

Extraction Method: Principal Axis Factoring.

Rotation Method: Varimax with Kaiser Normalization.a

a. Rotation converged in 7 iterations.

**Table 3:** Factor Analysis of Screening for Somatic Symptom Disorders-7(SOMS-7) in Patients with Anxious/Mood Disorder

**Title of Items**	**Factors**	
	**1**	**2**
1. Headache	0.466	
2. Stomachache		0.492
3. Lumbago		0.492
4. Pain in joints		0.649
5. Pain in hand, leg		0.651
6. Pain in chest	0.607	
7. Pain in anus		0.576
8. Pain during sexual intercourse	0.010	0.259
9. pain during urination	0.304	
10. Nausea	0.556	
11. Meteorism		0.643
12. Stomachache Anxiety	0.520	
13. Vomiting(not in pregnancy)	0.496	
14. Belching	0.114	0.218
15. Hiccups Burning in chest	0.304	
16. Inability to digest some foods		0.341
17. Inappetence	0.463	
18. Sour taste in mouth	0.602	
19. Mouth dryness	0.481	
20. Repeated diarrhea	0.198	0.267
21. Exit of fluids or moisture from anus		0.372
22. Repeated urination		0.376
23. Repeated Defecation		0.547
24. Palpitations	0.798	
25. Discomfort in Heart	0.711	
26. Sweating(hot, cold)	0.746	
27. Hot flashes	0.768	
28. Shortness of Breath without activity	0.756	
29. Gasping for Breath	0.801	
30. Excess Fatigue without activity	0.523	
31. Spots, color changes in skin	0.472	
32. Frigidity		0.331
33. Discomfort in Genital organs		0.562
34. Imbalance of movements	0.438	
35. Muscular paralysis	0.235	0.274
36. Trouble swallowing	0.525	
37. Loss of voice		0.310
38. Cease to urinate	0.361	
39. Imagining (seeing/ hearing unreal things)		0.373
40. Loss of sense of touch or pain		0.386
41. Shivering	0.547	
42. Diplopia	0.365	
43. Temporary Blindness	0.204	0.265
44. Temporary loss of hearing		0.399
45. Convulsion Attacks	0.037	0.136
46. Amnesia	0.285	0.027
47. Losing consciousness	0.123	-0.130
Number	79	82
Cronbach's alpha	0.910	0.810

Extraction Method: Principal Axis Factoring.

Rotation Method: Varimax with Kaiser Normalization.a

**Table 4:** Correlation between Screening for Somatic Symptom Disorders-7(SOMS-7) Factors and PHQ Scores in Normal Population

**Iranian healthy population**	**Factor**	**Pearson correlation coefficient**	**P-value**
PHQ somatic scale	First	0.659	0.001
	Second	0.341	0.001
	Third	0.081	0.236
	Fourth	0.256	0.001
PHQ depression scale	First	0.399	0.001
	Second	0.349	0.001
	Third	0.112	0.105
	Fourth	0.220	0.001
PHQ panic scale	First	0.535	0.001
	Second	0.166	0.017
	Third	0.269	0.001
	Fourth	0.051	0.471
PHQ anxiety scale	First	0.519	0.001
	Second	0.228	0.001
	Third	0.013	0.849
	Fourth	0.302	0.001
PHQ eating scale	First	0.244	0.001
	Second	0.224	0.001
	Third	0.098	0.154
	Fourth	0.186	0.007

**Table 5:** Correlation between Screening for Somatic Symptom Disorders-7(SOMS-7) Factors and PHQ Scores in Patients

**Anxiety/Mood Disorder Patients**	**Factor**	**Pearson Correlation Coefficient**	**P-value**
PHQ somatic questions	First	0.516	0.001
	Second	0.592	0.001
PHQ depression questions	First	0.304	0.010
	Second	-0.044	0.718
PHQ panic questions	First	0.187	0.125
	Second	-0.006	0.960
PHQ anxiety questions	First	0.140	0.248
	Second	0.212	0.078
PHQ eating questions	First	-0.005	0.967
	Second	0.156	0.198

**Table 6:** Differences of the Mean Score of SOMS-7 in Patients with Mood/Anxiety Disorder and Healthy Population

	**Number**	**Score of SOMS-7** **Mean ± Standard ** **Deviation**	**P-value**
Normal population	285	15.43±17.90	<0.001
Anxiety disorder patients	35	32.65±23.56	
Mood disorder patients	64	34.50±24.15	

## Discussion

Interviewing with psychiatric patients is the cornerstone of psychiatric diagnosis, however, to screen large populations and determine the effectiveness of therapeutic interventions, there is a need for precise assessment tools. Thus, the need for finding particular instruments for early diagnosis of SDD to lower health care costs and family pressure is elucidated.

Considering the above-mentioned and lacking a valid Persian Questionnaire to screen SSD and SFD, this study was conducted on an Iranian population. To determine factor structure and validation of SOMS-7, exploratory factor analysis was used in the general population. Four factors including (1) pain, cardiovascular and respiratory symptoms, (2) gastrointestinal and urologic symptoms, (3) neurological functioning symptoms and (4) musculoskeletal symptoms were found. All symptoms can be divided to 4 factors, and somatic symptoms disorders can be screened and diagnosed using the mentioned factors. Questions 21, 22, and 23 were candidates to be removed from the healthy population, and this might have been due to cultural differences of the Iranian population compared to the German population. Meanwhile, this questionnaire was divided into 2 categories for mood/anxiety disorder patients. Also, 8 questions were candidates to be removed again. Another study conducted by Wilma L. Zijlema et al. divided SOMS-7 questionnaire to 5 factors and reported this questionnaire as suitable for screening somatic symptoms disorder (13).

Test-retest assessment of the Persian SOMS-7 with a 2-week interval revealed a reliability of 0.86 in the healthy population and 0.70 in anxiety/mood disorder patients. This finding is comparable with that of a study which found SOMS-7 retest reliability of 0.76 in a period of 4 months and severity index of 0.71 (8). Another study reported 72-hour retest assessment of SOMS-7 and presented reliability of 0.85 and validity of 0.75(7). Few studies have been done using this questionnaire, but other questionnaires have been assessed including Composite International Diagnostic Interview (CIDI) with test-retest value of 0.74, 0.68, and 0.71 for somatization disorder, pain disorder, and hypochondriasis, respectively(14). Somatoform Disorders Schedule (SDS) Questionnaire was another that had a reliability of 0.76 (15).

In this study, reliability of 0.92- 0.94 was found. This Cronbach's alpha is somewhat similar to what was reported in other studies. Rief W et al. presented Cronbach's alpha of 0.92 (7).. Other questionnaires were reported to have high internal consistency. Thus, in term of reliability, SOMS-7 is as valuable as Symptom Questionnaire and SCL-90-R. Moreover, the internal consistency of SOMS-7 questionnaire is higher than what was reported for the Whiteley Index (0.80) and the Illness Attitude Scales (0.90) (8). Therefore, it can be concluded that SOMS-7 has acceptable validity for diagnosis of somatic symptom disorder in non-Iranian communities similar to what we found in this Persian version.

Another part of this study was to assess validity of SOMS-7. For this purpose, correlation of SOMS-7 scores with that of PHQ-15 was calculated. Four SOMS-7 subtypes were significantly correlated with PHQ subscales, including somatic, depression, panic, and anxiety. Based on factor analysis of SOMS-7 in patients with anxiety/mood disorders, the Persian version of SOMS-7 was categorized into 2 subtypes: (1) cardiovascular, respiratory and gastrointestinal symptoms, and (2) pain, musculoskeletal and neurological symptoms. These factors were associated with PHQ-15 scores as well. Findings of this study support those of Naz study (2016), which was conducted in Pakistan. They designed SSS questionnaire and found significant correlations in conversion, pain, hypochondriasis, and body dysmorphic syndromes with SOMS scores (16). In the study of Zijlema WL et al., this correlation between SOSM-7 and SCL-R was 0.76, and the highest correlation was seen between SOMS-7 and PHQ-15 (13), which is similar to the present study. Patients with anxiety/mood disorder obtained significantly higher scores on SOMS-7 compared to healthy population, which could indicate another validity index of the Persian SOMS-7 version. 

Discriminant analysis was conducted to determine cut-off point score and its sensitivity and specificity. Findings showed that obtaining the score of 15.5 and above can predict somatic symptom disorder with sensitivity of 77% and specificity of 66%. SOMS-7 questionnaire had sensitivity of 98% and specificity of 63% in another study (7). These differences may be due to the diversity of assessed populations in the current study, in which we compared healthy population with anxiety/mood disorder patients, while in the study of Hiller W et al., healthy population was compared with somatoform disorder patients (7). 

## Limitation

The most important limitation of this study was lack of cut-off determination of the Persian version of SOMS-7 based on patients with SSD. These cut-off points and their sensitivity and specificity can be used to discriminate between the general population and patients with mood/anxiety disorder based on somatic symptoms in mood/anxiety disorder. Thus, we suggest that future studies consider SOMS-7 discriminant validity based on cut-off point and sensitivity and specificity between the general population and patients with somatic symptoms disorder.

## Conclusion

Findings of this study indicate that the Persian version of SOMS-7 has suitable validity and reliability for screening healthy population for abnormal somatic symptoms complaints. The findings of this study also indicated that this tool can be used to measure the effects of psychological interventions.
